# *Ginkgo biloba* extract for dizziness-related symptoms in central neurological disorders: a systematic review and meta-analysis

**DOI:** 10.3389/fneur.2026.1860538

**Published:** 2026-06-18

**Authors:** Young-Hyo Jeon, Jeong Ah Kim, Dong Yoon Kang, Seunghyun Lee, Nam-Kyong Choi, Ji-Yun Park

**Affiliations:** 1 Department of Health Convergence, College of Science and Industry Convergence at Ewha Womans University, Seoul, Republic of Korea; 2 Department of Preventive Medicine, Ulsan University Hospital, University of Ulsan College of Medicine, Ulsan, Republic of Korea; 3 Department of Neurology, Ulsan University Hospital, University of Ulsan College of Medicine, Ulsan, Republic of Korea; 4 Department of Industrial Pharmaceutical Science, College of Pharmacy, Ewha Womans University, Seoul, Republic of Korea

**Keywords:** cerebrovascular disorders, dizziness, *Ginkgo biloba*, meta-analysis, plant extracts, vertigo

## Abstract

**Background and purpose:**

Dizziness associated with central neurological disorders—broadly defined as dizziness or vertigo attributable to central nervous system pathology affecting central vestibular processing—is a clinically challenging and heterogeneous condition with limited treatment options. *Ginkgo biloba* extract—through its microcirculatory, neuroprotective, and anti-inflammatory mechanisms—represents a biologically plausible intervention. However, its efficacy in this setting has not been comprehensively established. We evaluated the efficacy and safety of *Ginkgo biloba* extract through a systematic review and meta-analysis of randomized controlled trials (RCTs).

**Methods:**

Nine international and Korean databases were searched from January 1974 through November 2025. Studies were eligible if they were RCTs enrolling adults aged 18 years or older with cerebrovascular disease, neurodegenerative disease, or central vestibular dysfunction who had dizziness, vertigo, or balance-related symptoms or relevant outcome assessments. Cochrane RoB 2.0 tool and certainty of evidence was rated using the Assessment, Development and Evaluations (GRADE) approach. Prespecified subgroup analyses by underlying etiology and intervention type, together with leave-one-out sensitivity analyses, were performed to explore heterogeneity.

**Results:**

Nine RCTs (*N* = 2,394) were included; participants were predominantly drawn from dementia populations (71.6%), with smaller contributions from cerebral arteriosclerosis (23.0%) and vertebrobasilar or posterior circulation disorders (5.4%). *Ginkgo biloba* significantly reduced dizziness/vertigo severity on the 11-point box scale (MD − 0.76, 95% CI − 1.35 to −0.18; *p* = 0.01) and VAS (SMD − 0.38, 95% CI − 0.58 to −0.19; *p* = 0.0001). Moderate-certainty evidence suggested improvements in functional outcomes and quality of life, including the Alzheimer’s Disease Activities of Daily Living International Scale (MD = −0.17, 95% CI: −0.22 to −0.13) and the Dementia Quality of Life – Proxy (MD = 2.00, 95% CI: 0.85 to 3.15). The intervention was generally well tolerated, with significantly lower risks of angina pectoris (OR 0.51, 95% CI 0.31 to 0.85) and tinnitus (OR 0.37, 95% CI 0.22 to 0.63) and no significant increase in other adverse events.

**Conclusion:**

*Ginkgo biloba* extract may reduce dizziness severity and improve daily functioning in patients with central neurological disorders accompanied by dizziness or vertigo, with a favorable safety profile. However, given the small number of eligible trials, substantial clinical and statistical heterogeneity, and the predominance of dementia-derived data, these findings should be interpreted with caution. Well-designed RCTs in clearly defined central vestibular populations, ideally confirmed by neuroimaging or vestibular testing, are needed to confirm these results.

**Systematic review registration:**

https://www.crd.york.ac.uk/prospero/display_record.php?ID=CRD420251229692, PROSPERO: CRD420251229692.

## Introduction

Dizziness is a common and disabling neurological symptom affecting 15–35% of adults and up to 40% of those over 65 years of age (1, 2). Among its subtypes, dizziness associated with central neurological disorders—arising from pathology in the brainstem, cerebellum, or cerebral cortex—is particularly burdensome because it tends to be more persistent, diagnostically challenging, and more strongly associated with serious neurological disease than peripheral vestibular disorders. Dizziness related to central neurological disorders accounts for approximately 20–25% of dizziness presentations and contributes disproportionately to morbidity, falls, functional decline, and healthcare utilization (3, 4). Such dizziness reflects dysfunction within central vestibular pathways, including the vestibular nuclei, cerebellum, thalamus, and vestibular cortex. In this review, dizziness associated with central neurological disorders refers broadly to dizziness or vertigo attributable to central nervous system pathology affecting central vestibular processing, including cerebrovascular and neurodegenerative disorders. Common etiologies include vertebrobasilar insufficiency, cerebral small vessel disease, posterior fossa or brainstem infarctions, and neurodegenerative disorders such as Alzheimer’s disease and vascular dementia (5, 6). In these conditions, microvascular ischemia, neuroinflammation, oxidative stress, and disruption of neurotransmitter systems collectively impair central vestibular integration and compensation, producing persistent and often treatment-refractory dizziness. However, the concept of “central vertigo” encompasses a clinically heterogeneous spectrum, ranging from primary central vestibular disorders to secondary dizziness/vertigo associated with cognitive impairment or gait instability in neurodegenerative disease. This heterogeneity complicates both diagnosis and therapeutic evaluation. Unlike peripheral vestibular disorders, which tend to resolve spontaneously or respond well to repositioning maneuvers, dizziness associated with central neurological disorders typically requires active pharmacological management targeting the underlying cerebrovascular or neurodegenerative pathology. Current pharmacological options for dizziness associated with central neurological disorders are limited. Vestibular suppressants such as antihistamines and benzodiazepines provide short-term relief but impede central vestibular compensation and worsen cognitive function—effects particularly undesirable in elderly patients with neurodegenerative comorbidities (7). Betahistine, widely used for Ménière’s disease, has limited evidence in centrally mediated dizziness. Standard cerebrovascular therapies such as antiplatelet agents, statins, and antihypertensives address underlying risk factors but do not directly ameliorate dizziness symptoms (8). This unmet need highlights the importance of identifying agents with dual neuroprotective and vestibulo-symptomatic benefit.

*Ginkgo biloba* extract (standardized as EGb 761®; 24% flavonoid glycosides, 6% terpene lactones including ginkgolides A, B, C and bilobalide) has multiple pharmacological mechanisms relevant to dizziness associated with central neurological disorders. Ginkgolides potently inhibit platelet-activating factor (PAF), reducing platelet aggregation and improving cerebral microcirculation, thereby directly addressing the vertebrobasilar ischemic component of centrally mediated dizziness (9). Flavonoid glycosides and bilobalide scavenge reactive oxygen species, reduce neuroinflammation (suppressing NF-κB, IL-6, TNF-*α*), and exert neuroprotective effects against ischemic injury (10, 11). Additionally, *Ginkgo biloba* modulates serotonin, dopamine, and acetylcholine neurotransmitter systems implicated in central vestibular processing, and enhances cerebral blood flow velocity. These properties suggest that *Ginkgo biloba* may have therapeutic relevance in conditions associated with central vertigo, although its effects on vestibular symptoms per se remain incompletely defined. Approved in France in 1974, EGb 761® is used in numerous countries for cognitive decline, dementia, and cerebrovascular disease (12).

Several randomized controlled trials (RCTs) have evaluated *Ginkgo biloba* extract in populations with central neurological disorders accompanied by dizziness/vertigo-related conditions—including cerebral arteriosclerosis, vertebrobasilar ischemia, dementia (Alzheimer’s disease [AD]/vascular dementia [VaD]), and post-ischemic stroke—reporting significant reductions in dizziness/vertigo severity and improvements in cerebral hemodynamics and functional outcomes. A 12-week multicenter RCT found EGb 761® 240 mg/day to be at least as effective as betahistine 32 mg/day for vertigo (13). However, the available evidence is heterogeneous in terms of patient populations, outcome measures, and study designs, and to date, no comprehensive and methodologically rigorous systematic review and meta-analysis has specifically addressed the efficacy and safety of *Ginkgo biloba* for dizziness/vertigo symptoms occurring in the context of central neurological disorders. Therefore, this study aimed to evaluate the efficacy and safety of *Ginkgo biloba* extract in adult patients with central nervous system disorders accompanied by dizziness/vertigo symptoms through a systematic review and meta-analysis of RCTs.

## Methods

The protocol for this systematic review and meta-analysis was prospectively registered in PROSPERO (CRD420251229692), and the study was conducted in accordance with the PRISMA 2020 guidelines (14). In this study, we included randomized controlled trials involving central neurological disorders in which dizziness-, vertigo-, balance-related symptoms, or related functional outcomes were assessed.

### Search strategy

A systematic literature search was conducted across nine databases, including five international databases (PubMed, Web of Science, EMBASE, CINAHL, and Cochrane CENTRAL) and four Korean databases (KMbase, KoreaMed, RISS, and ScienceON). The search covered studies published from January 1, 1974, the year *Ginkgo biloba* extract received its first regulatory approval in France, through November 30, 2025 (15). To minimize the risk of missing eligible studies, manual searches were additionally performed. Forward citation tracking was conducted to identify studies that cited the included literature, and backward citation searches were performed by reviewing the reference lists of included studies to ensure search completeness. The search terms included “vertigo,” “dizziness,” “vestibular disease,” “postural balance,” “ginkgo*,” “*Ginkgo biloba*,” “gingko*,” “ginko*,” “EGb 761,” and “random*.” Detailed search strategies for each database are provided in the Supplementary Table S1.

### Eligibility criteria

#### Inclusion criteria

Studies were eligible for inclusion if they met the following PICO-SD (Population, Intervention, Comparison, Outcome, and Study Design) criteria: (1) Population: patients aged 18 years or older with central neurological disorders who had dizziness, vertigo, or balance-related symptoms or outcome assessments. For the purposes of this review, central vertigo was broadly defined as dizziness or vertigo attributable to central nervous system pathology affecting central vestibular processing, including cerebrovascular disorders, vertebrobasilar insufficiency, cerebral arteriosclerosis, post-stroke states, and neurodegenerative disorders such as Alzheimer’s disease or vascular dementia; (2) Intervention: administration of *Ginkgo biloba* extract as monotherapy or as an add-on to standard care; (3) Comparison: placebo or other treatments administered alone, or placebo or other treatments added to standard care; (4) Outcome: clinical efficacy and safety of the intervention for vertigo-related symptoms. Representative outcomes included clinical response rate, which was defined as the proportion of patients rated as improved or above on a four-category ordinal scale (e.g., remarkably improved, improved, unchanged, or aggravated), subjective symptom measures and objective functional measures related to vertigo, and global quality-of-life scales; (5) Study design: only RCTs were eligible for inclusion.

#### Exclusion criteria

Studies were excluded if they met any of the following criteria: (1) non-randomized study designs; (2) duplicate publications; (3) unavailability of full text; (4) language other than English or Korean; (5) non-human studies; (6) non-peer-reviewed publications; (7) absence of ginkgo biloba as a study intervention; (8) exclusive focus on peripheral vertigo; and (9) inaccurate, ambiguously described, or incompletely reported data.

### Study selection and data extraction

Study selection was performed independently by two reviewers (Y-HJ and JK). Title and abstract were screened in the first stage, followed by full-text review of potentially eligible studies. Data extraction was conducted independently by two reviewers using a standardized extraction form. Extracted information included study characteristics (authors, publication year, country, study design), sample size, characteristics of the intervention and comparator groups, and outcome measures. Any disagreements at either stage were resolved through discussion and consensus, with a third reviewer (J-YP) consulted when consensus could not be reached.

### Risk of bias assessment

Risk of bias was assessed independently by two reviewers (Y-HJ and JK) using the Cochrane Risk of Bias 2 (RoB 2.0) tool. It evaluates five domains: the randomization process, deviations from intended interventions, missing outcome data, measurement of the outcome, and selection of the reported result (16). Based on these domains, each study was assigned an overall risk of bias judgment of “Low,” “Some concerns,” or “High.” Discrepancies between reviewers were resolved through discussion, with adjudication by a third reviewer (J-YP) where necessary.

### Statistical analysis

Meta-analyses were performed using RevMan 5.4. For dichotomous outcomes, pooled effect estimates were expressed as odds ratios (ORs) with 95% confidence intervals (CIs). For continuous outcomes, mean differences (MDs) with 95% CIs were calculated. Standardized mean differences (SMDs) were used when outcome measures differed across studies, including instances where the same instrument was administered using different scale ranges. Heterogeneity among studies was evaluated using Higgins’ *I*^2^ statistic. A fixed-effects model was applied using the Mantel–Haenszel method when heterogeneity was low (I^2^ < 50%) (17). A random-effects model was applied using the DerSimonian–Laird method when heterogeneity was substantial (I^2^ ≥ 50%).

Sensitivity analyses were conducted by sequentially excluding individual studies to assess the robustness of pooled estimates. When 10 or more eligible RCTs were available, a funnel plot was constructed to evaluate the potential influence of publication bias (18). Subgroup analyses were prespecified to examine differences in treatment effects according to (i) patient population/underlying etiology, (ii) intervention type, and (iii) the mode of administration: (a) monotherapy versus (b) add-on to standard care.

### Evidence certainty assessment

The certainty of evidence for each outcome was assessed using the Grading of Recommendations, Assessment, Development and Evaluations (GRADE) approach (19). Evidence was rated across five domains, including risk of bias, inconsistency, indirectness, imprecision, and publication bias, and classified as “High,” “Moderate,” “Low,” or “Very low” (20). Assessments were performed independently by two reviewers (Y-HJ and JK), with disagreements resolved by a third reviewer (J-YP).

## Results

### Literature search and screening

**Figure 1:**
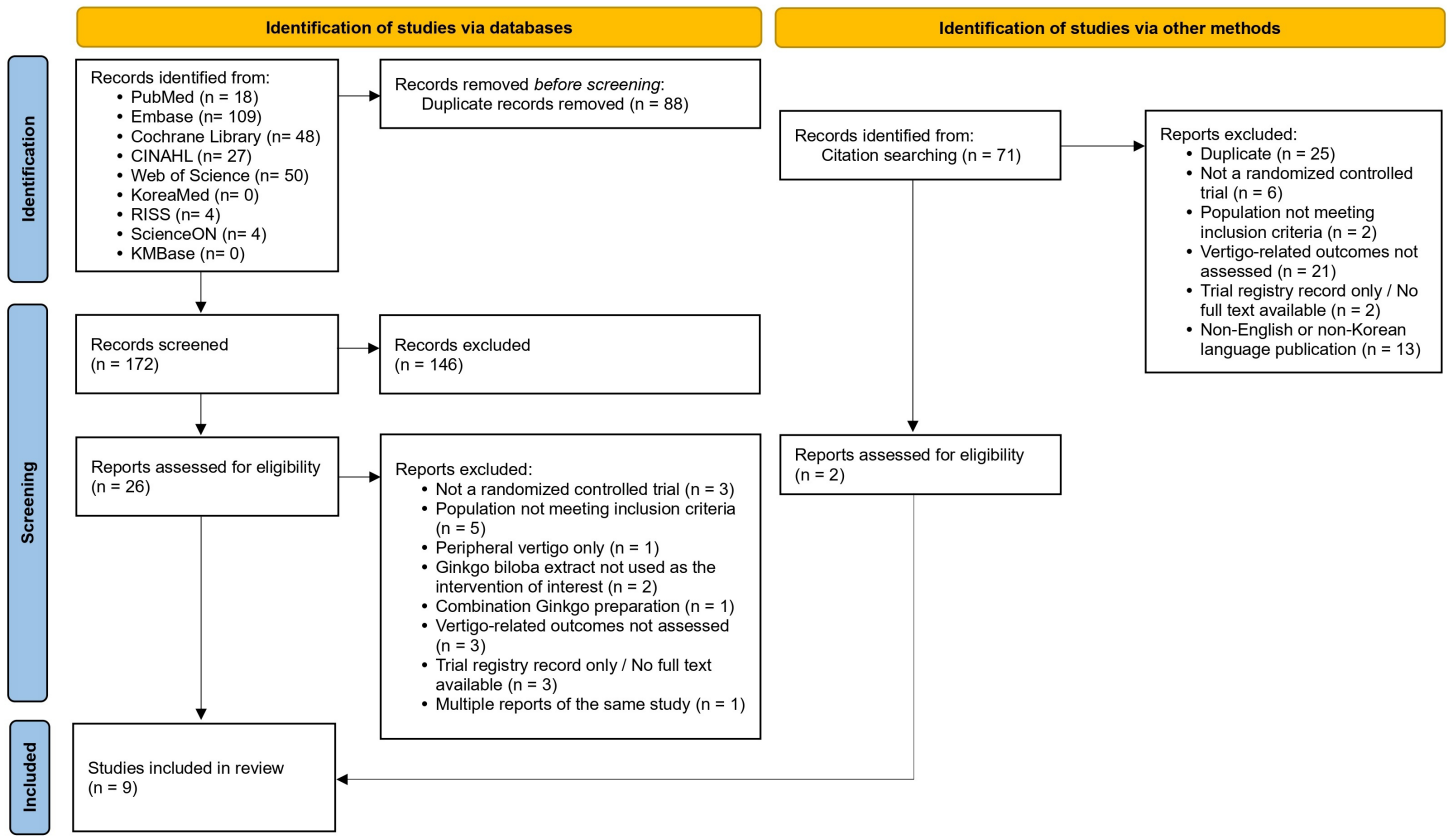
Flow diagram of study selection.

Database searching identified 260 records from PubMed (*n* = 18), Embase (*n* = 109), Cochrane Library (*n* = 48), CINAHL (*n* = 27), Web of Science (*n* = 50), KoreaMed (*n* = 0), RISS (*n* = 4), ScienceON (*n* = 4), and KMBase (*n* = 0). After removal of 88 duplicates, 172 records were screened, of which 26 proceeded to full-text review. Of these, 19 were excluded for the following reasons: not a randomized controlled trial (*n* = 3), population not meeting inclusion criteria (*n* = 5), peripheral vertigo only (*n* = 1), ginkgo biloba extract not used as the intervention of interest (*n* = 2), combination ginkgo preparation (*n* = 1), vertigo-related outcomes not assessed (*n* = 3), trial registry record only or no full text available (*n* = 3), and multiple reports of the same study (*n* = 1). Citation searching yielded an additional 71 records, of which 2 underwent full-text review. Ultimately, 9 studies were included in the review (Figure 1) (21–29).

### Characteristics of included studies

The nine studies were published between 1995 and 2024, including a total of 2,394 participants (treatment group, *n* = 1,284; control group, *n* = 1,110). Study populations comprised patients with central vertigo or dizziness due to cerebral circulatory disorders, vascular vestibular disorders, Alzheimer’s disease or vascular dementia, cerebral arteriosclerosis, or vertebrobasilar ischemia. The included populations were predominantly derived from dementia cohorts (71.6%), with smaller contributions from cerebral arteriosclerosis (23.0%) and vertebrobasilar or posterior circulation disorders (5.4%), reflecting the clinical breadth of dizziness associated with central neurological disorders. For subgroup analyses according to underlying etiology, studies were categorized into cerebrovascular etiology subgroup and dementia subgroup. In addition, because different ginkgo biloba formulations were used across studies, subgroup analyses according to intervention type were performed for studies using EGb 761® as the intervention. Eight studies administered ginkgo biloba extract as monotherapy, while one study employed it as an add-on to betahistine dihydrochloride. Outcome measures varied across studies. Outcomes reported by two or more studies included clinical response rate, the 11-point box scale for dizziness, Visual Analogue Scale (VAS), Dizziness Handicap Inventory (DHI), Alzheimer’s Disease Activities of Daily Living International Scale (ADL-IS), Dementia Quality of Life—Proxy version (DEMQOL–Proxy), and adverse events. Detailed characteristics of included studies are summarized in Table 1.

**Table 1:** Characteristics of the included studies.

Study	Patients	Treatment	Control	No. of patients	Duration (weeks)	*Ginkgo biloba* dose (mg)	Age(y) (mean [SD])	Sex male (%)	Outcome
Kim et al. (21)	Outpatients with central vertigo due to cerebral circulatory disorders	Ginkgo leaf dried extract (Tanamin®)	Placebo	Total: 57 T: 31C: 26	24	40 three times daily	T: 60.8C: 55.5	T: 13(41.9) C: 9(34.6)	①㉒
Cesarani et al. (22)	Patients with vertigo, dizziness, or both caused by vascular vestibular disorders	EGb 761	Betahistine dihydrochloride	Total: 37 T: 20C: 17	12	80 twice daily	T: 64.55 (5.07) C: 63.59 (4.78)	T: 5(25.0) C: 5(29.4)	①②③④ ⑤⑥⑦⑧㉒
Schneider et al. (23)	Outpatients with probable Alzheimer’s disease	EGb 761	Placebo	Total: 513 T (120 mg): 169 T (240 mg): 170C: 174	26	T (120 mg): 60 twice daily T (240 mg): 120 twice daily	T (120 mg): 78.6 (7.0) T (240 mg): 78.1 (7.0) C: 77.5 (7.4)	T (120 mg): 85 (50.3) T (240 mg): 74 (43.5) C: 84 (48.3)	⑨㉒
Napryeyenko et al. (24)	Patients with mild to moderate Alzheimer’s disease or vascular dementia	EGb 761	Placebo	Total: 395 T: 198C: 197	22	120 twice daily	T: 65 (8) C: 63 (8)	T: 55 (27.8) C: 55 (27.9)	⑨⑩㉒
Ihl et al. (25)	Patients with mild to moderate Alzheimer’s disease or vascular dementia	EGb 761	Placebo	Total: 404 T: 202C: 202	24	240 once daily	T: 65 (10) C: 65 (9)	T: 63 (31.2) C: 69 (34.2)	⑨⑪⑫㉒
Herrschaft et al. (26)	Patients with mild to moderate Alzheimer’s disease or vascular dementia	EGb 761	Placebo	Total: 402 T: 200C: 202	24	240 once daily	T: 65.1 (8.8) C: 64.9 (9.4)	T: 61 (30.5) C: 62 (30.7)	⑨⑪⑫㉒
Rina et al. (27)	Patients with dizziness caused by cerebral arteriosclerosis	GBE 50	Naoxinqing	Total: 380 T: 191C: 189	6	40 three times daily	T: 59 (6) C: 59 (6)	T: 84 (44.0) C: 72 (38.1)	①⑬⑭⑮⑯
Heide et al. (28)	Patients with central vestibular vertigo due to vertebrobasilar transient ischemic attacks or infarctions	EGb 761	Placebo	Total: 36 T: 18C: 18	24	120 twice daily	T: 63C: 55	T: 11 (61.1) C: 15 (83.3)	⑭⑰⑱⑲㉒
Li and Cao (29)	Patients with cerebral arteriosclerosis exhibiting prominent dizziness symptoms	Betahistine dihydrochloride + GBE injection	Betahistine dihydrochloride	Total: 170 T: 85C: 85	2	17.5 once daily	T: 63.64 (5.95) C: 64.81 (5.05)	T: 49 (57.6) C: 42 (49.4)	⑬⑳㉑

T, treatment group; C, control group; ① Clinical response rate; ② Romberg test; ③ Babinski–Weil test; ④ Horizontal random saccades; ⑤ Horizontal smooth pursuit; ⑥ Optokinetic nystagmus; ⑦ Vestibulo-ocular reflex (VOR); ⑧ Visuovestibular reflex (VVOR); ⑨ 11-point box scale for dizziness; ⑩ Gottfries–Bråne–Steen Activities of Daily Living subscale (GBS-ADL); ⑪ Alzheimer’s disease activities of daily living international scale (ADL-IS); ⑫ Dementia quality of life—proxy version (DEMQOL-Proxy); ⑬ Dizziness Handicap Inventory (DHI); ⑭ Visual Analogue Scale (VAS); ⑮ University of California vertigo questionnaire (UCLA-DQ); ⑯ Single-item symptom score of traditional Chinese Medicine (TCM); ⑰ Physician’s Global Rating of Change (PGRC); ⑱ Vertigo score; ⑲ Direct current (DC) electronystagmography parameters; ⑳ Functional scale for cervical spondylosis of vertebral artery type (FS-CSA); ㉑ Barthel index (BI); ㉒ Adverse events.

### Risk of bias assessment

**Figure 2:**
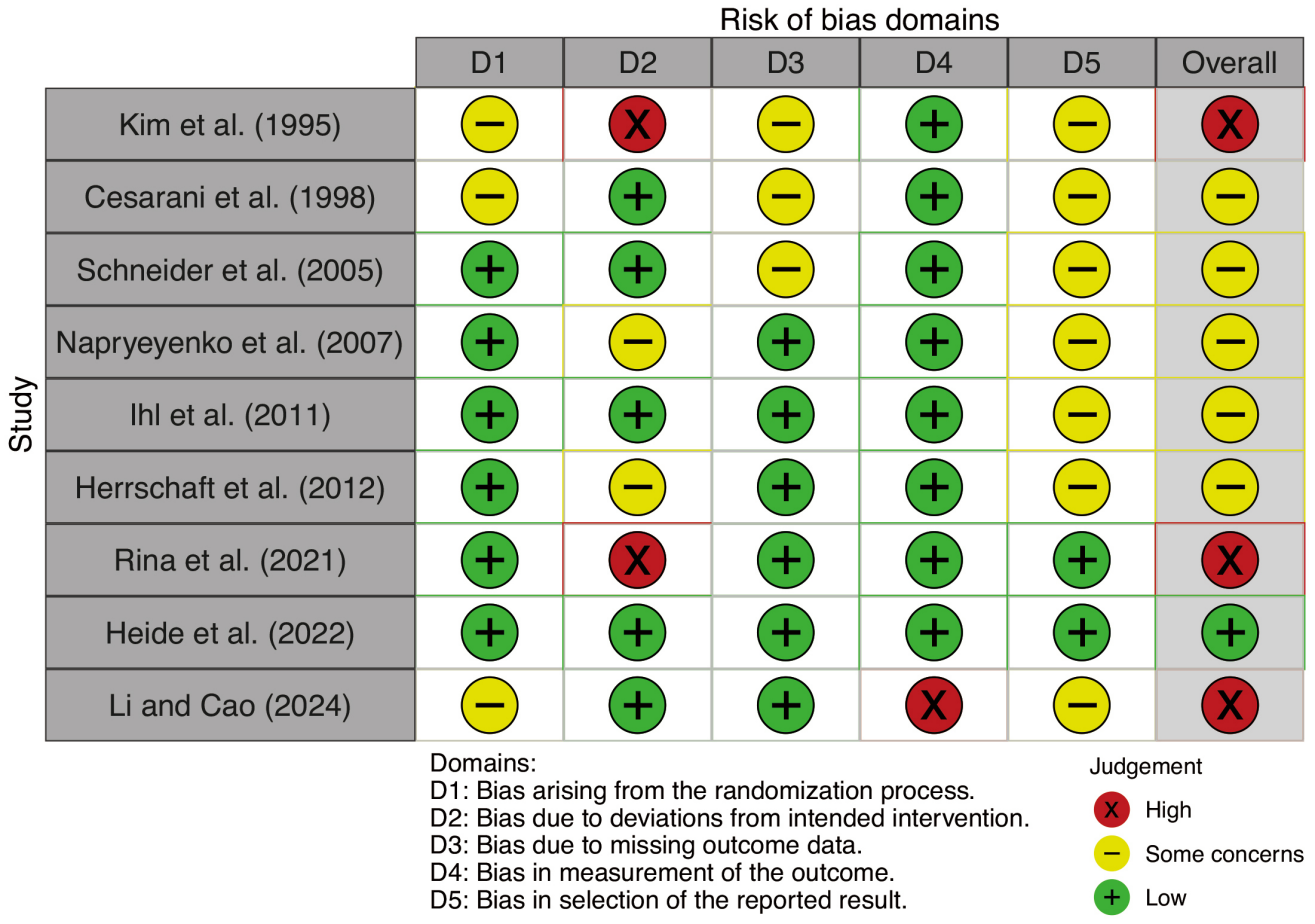
Risk of bias (RoB 2.0) graph.

**Figure 3:**
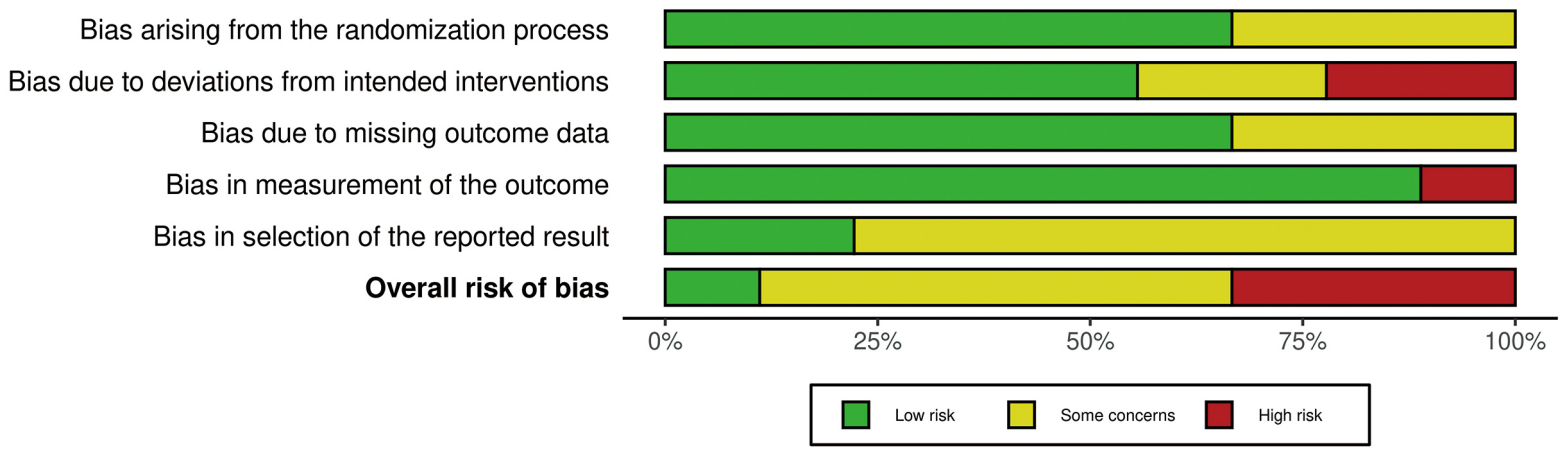
Risk of bias (RoB 2.0) summary.

Nine studies were assessed using RoB 2.0 (Figures 2, 3). For the randomization process, most studies were rated as low risk. Regarding deviations from intended interventions, two studies were rated as high risk, with the remaining studies rated as low risk or some concerns. Missing outcome data was rated as low risk in most studies. For measurement of the outcome, one study was rated as high risk. Selection of the reported result was rated as some concerns in most studies, suggesting insufficient pre-registration or protocol reporting. Overall, one study was rated as low risk, five as some concerns, and three as high risk.

### Efficacy

Efficacy outcomes were reported in 7 of 9 studies (21, 22, 24–28). Although DHI was reported in two studies (27, 29), one study (29) did not provide sufficient numerical data for meta-analysis and was therefore excluded from the pooled analysis.

### Vertigo-related outcomes

#### Clinical response rate

**Figure 4:**
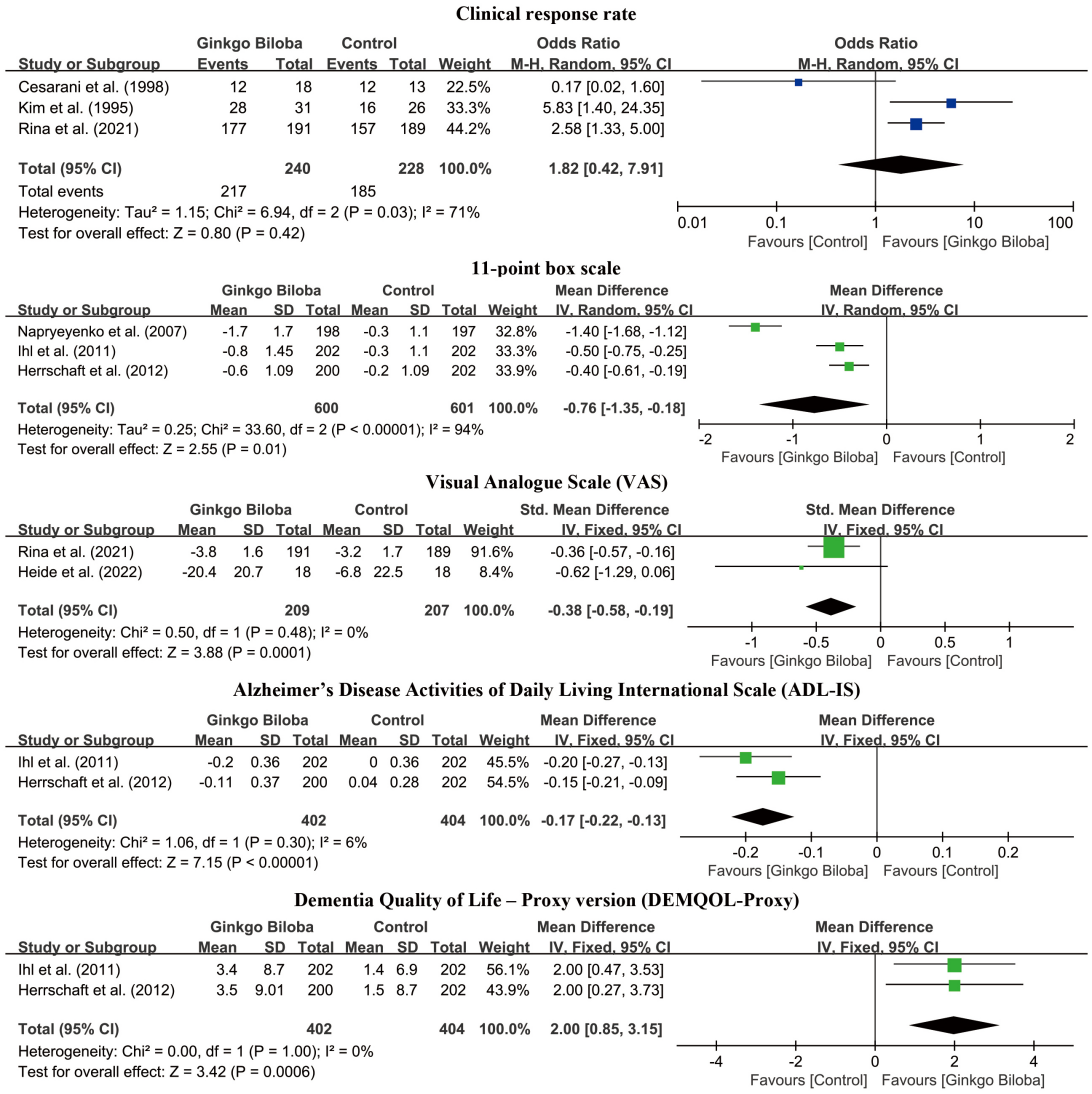
Meta-analysis of the effects of ginkgo biloba on dizziness improvement and quality of life.

Three studies (21, 22, 27) including 468 participants with cerebrovascular disorders reported clinical response rate, defined as the proportion of patients achieving improvement or better on a four-category ordinal scale. Heterogeneity was substantial (*I*^2^ = 71%), and a random-effects model was applied. No statistically significant difference was found between ginkgo biloba and control groups (OR = 1.82, 95% CI: 0.42 to 7.91) (Figure 4).

#### 11-point box scale

Three studies in dementia populations (24–26) including 1,201 participants assessed dizziness severity using the 11-point box scale, on which higher scores indicate greater symptom severity. Given substantial heterogeneity (*I*^2^ = 94%), a random-effects model was used. *Ginkgo biloba* significantly reduced dizziness scores compared to control (MD = −0.76, 95% CI: −1.35 to −0.18) (Figure 4).

#### Visual analogue scale (VAS)

Dizziness severity was measured using the VAS, ranging from 0 (no dizziness) to 10 (extreme dizziness), and was reported in two studies (27, 28) including 416 participants with cerebrovascular disorders. Because the included studies used different VAS scale ranges (0–10 and 0–100), SMD was used as the effect size measure. Heterogeneity was low (*I*^2^ = 0%), and a fixed-effects model was applied. *Ginkgo biloba* significantly reduced VAS scores compared to control (SMD = −0.38, 95% CI: −0.58 to −0.19) (Figure 4).

### Quality of life outcomes

#### Alzheimer’s disease activities of daily living international scale (ADL-IS)

Activities of daily living were evaluated using the ADL-IS, on which lower scores indicate better functional status, across two studies in dementia populations (25, 26) including 806 participants. Heterogeneity was low (*I*^2^ = 6%), and a fixed-effects model was applied. *Ginkgo biloba* significantly improved ADL-IS scores compared to control (MD = −0.17, 95% CI: −0.22 to −0.13) (Figure 4).

#### Dementia quality of life—proxy version (DEMQOL-proxy)

Health-related quality of life was assessed using the DEMQOL-Proxy, on which higher scores reflect better quality of life in in dementia populations (25, 26). Heterogeneity was negligible (*I*^2^ = 0%), and a fixed-effects model was accordingly applied. *Ginkgo biloba* significantly improved DEMQOL-Proxy scores compared to control (MD = 2.00, 95% CI: 0.85 to 3.15) (Figure 4).

### Safety

#### Adverse events

**Figure 5:**
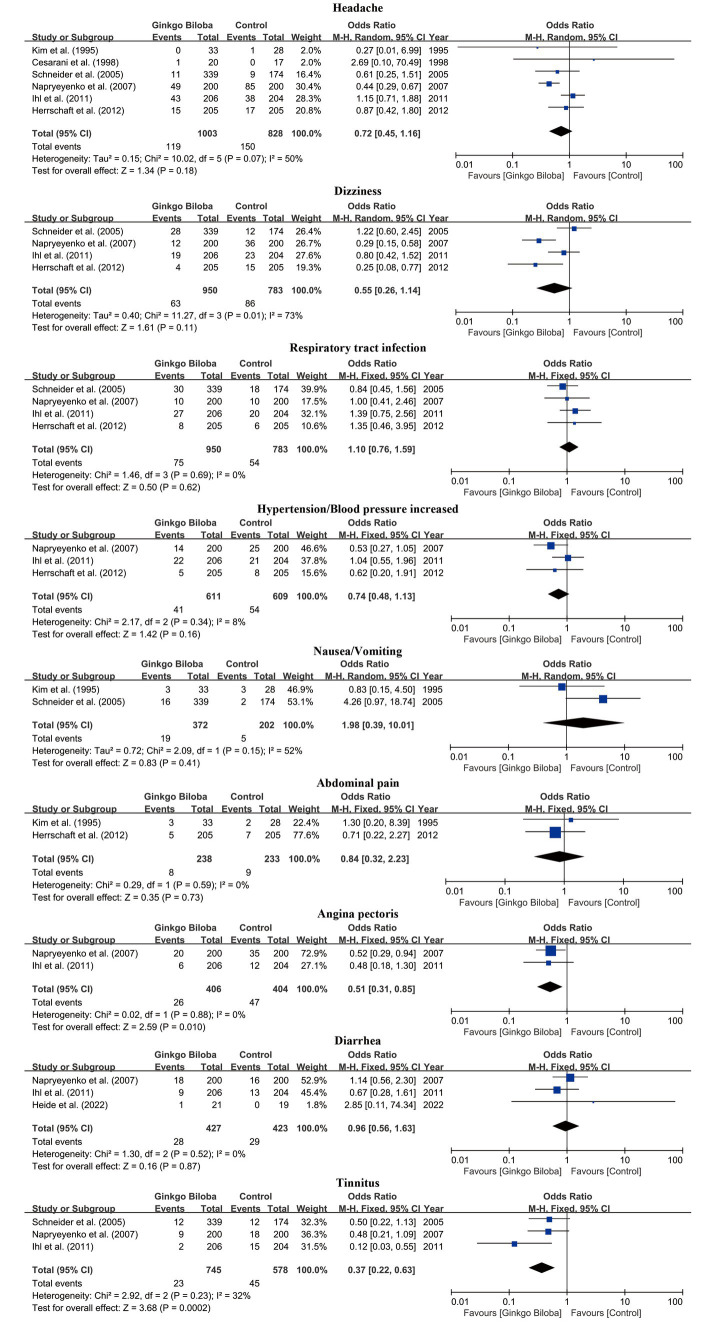
Meta-analysis of the adverse events of ginkgo biloba.

Adverse events were reported in 7 of the 9 included studies (21–26, 28). To enable pooled analysis, only adverse events reported in two or more studies were included. Among the adverse events assessed, angina pectoris and tinnitus occurred significantly less frequently in the ginkgo biloba group compared to control (OR = 0.51, 95% CI: 0.31 to 0.85, OR = 0.37, 95% CI: 0.22 to 0.63, respectively) (Figure 5). No statistically significant between-group differences were observed for headache (OR = 0.72, 95% CI: 0.45 to 1.16), dizziness (OR = 0.55, 95% CI: 0.26 to 1.14), respiratory tract infection (OR = 1.10, 95% CI: 0.76 to 1.59), hypertension/blood pressure increased (OR = 0.74, 95% CI: 0.48 to 1.13), nausea/vomiting (OR = 1.98, 95% CI: 0.39 to 10.01), abdominal pain (OR = 0.84, 95% CI: 0.32 to 2.23), or diarrhea (OR = 0.96, 95% CI: 0.56 to 1.63).

#### Certainty of evidence

The certainty of evidence for all outcomes was assessed using the GRADE approach and is summarized in Table 2. For efficacy outcomes, VAS was supported by moderate-certainty evidence, while the 11-point box scale was rated as low certainty due to heterogeneity and indirectness, as the outcome was assessed as a secondary measure in dementia populations. ADL-IS and DEMQOL-Proxy were also rated as moderate-certainty evidence, with indirectness downgraded because these outcomes were evaluated as secondary measures in dementia populations. Clinical response rate was rated as very low certainty, primarily due to very serious risk of bias and imprecision. For safety outcomes, angina pectoris was supported by moderate-certainty evidence, and tinnitus by low-certainty evidence. The remaining adverse events were rated as very low certainty, largely due to serious risk of bias, inconsistency, indirectness, or imprecision.

**Table 2:** Grading of recommendations assessment, development, and evaluation (GRADE) evidence profile.

Certainty assessment						No. of patients		Effect		Certainty
No. of studies	Risk of bias	Inconsistency	Indirectness	Imprecision	Other considerations	Ginko biloba	Control	Relative 95% CI)	Absolute (95% CI)	
Efficacy										
Clinical response rate										
3	Very serious	Serious	Not serious	Very serious	None	217/240 (90.4%)	185/228 (81.1%)	OR 1.82 (0.42 to 7.91)	75 more per 1,000 (from 168 fewer to 160 more)	⨁◯◯◯ Very low
11-point box scale										
3	Not serious	Serious	Serious	Not serious	None	600	601	—	MD 0.76 lower 1.35 lower to 0.18 lower)	⨁⨁◯◯ Low
Visual analogue scale (VAS)										
2	Serious	Not serious	Not serious	Not serious	None	209	207	—	SMD 0.38 SD lower (0.58 lower to 0.19 lower)	⨁⨁⨁◯ Moderate
Alzheimer’s disease activities of daily living international scale (ADL-IS)										
2	Not serious	Not serious	Serious	Not serious	None	402	404	—	MD 0.17 lower (0.22 lower to 0.13 lower)	⨁⨁⨁◯ Moderate
Dementia quality of life—proxy version (DEMQOL-Proxy)										
2	Not serious	Not serious	Serious	Not serious	None	402	404	—	MD 2 higher (0.85 higher to 3.15 higher)	⨁⨁⨁◯ Moderate
Safety										
Headache										
6	Serious	Serious	Serious	Serious	None	119/1003 (11.9%)	150/828 (18.1%)	OR 0.72 (0.45 to 1.16)	44 fewer per 1,000 (from 91 fewer to 23 more)	⨁◯◯◯ Very low
Dizziness										
4	Not serious	Serious	Serious	Serious	None	63/950 (6.6%)	86/783 (11.0%)	OR 0.55 (0.26 to 1.14)	46 fewer per 1,000 (from 79 fewer to 13 more)	⨁◯◯◯ Very low
Respiratory tract infection										
4	Serious	Not serious	Serious	Serious	None	75/950 (7.9%)	54/783 (6.9%)	OR 1.10 (0.76 to 1.59)	6 more per 1,000 (from 16 fewer to 36 more)	⨁◯◯◯ Very low
Hypertension/blood pressure increased										
3	Serious	Not serious	Serious	Serious	None	41/611 (6.7%)	54/609 (8.9%)	OR 0.74 (0.48 to 1.13)	22 fewer per 1,000 (from 44 fewer to 10 more)	⨁◯◯◯ Very low
Nausea/vomiting										
2	Serious	Serious	Serious	Very serious	None	19/372 (5.1%)	5/202 (2.5%)	OR 1.98 (0.39 to 10.01)	23 more per 1,000 (from 15 fewer to 178 more)	⨁◯◯◯ Very low
Abdominal pain										
2	Serious	Not serious	Serious	Very serious	None	8/238 (3.4%)	9/233 (3.9%)	OR 0.84 (0.32 to 2.23)	6 fewer per 1,000 (from 26 fewer to 44 more)	⨁◯◯◯ Very low
Angina pectoris										
2	Not serious	Not serious	Serious	Not serious	None	26/406 (6.4%)	47/404 (11.6%)	OR 0.51 (0.31 to 0.85)	53 fewer per 1,000 (from 77 fewer to 16 fewer)	⨁⨁⨁◯ Moderate
Diarrhea										
3	Not serious	Not serious	Serious	Very serious	None	28/427 (6.6%)	29/423 (6.9%)	OR 0.96 (0.56 to 1.63)	3 fewer per 1,000 (from 29 fewer to 39 more)	⨁◯◯◯ Very low
Tinnitus										
3	Serious	Not serious	Serious	Not serious	None	23/745 (3.1%)	45/578 (7.8%)	OR 0.37 (0.22 to 0.63)	48 fewer per 1,000 (from 60 fewer to 27 fewer)	⨁⨁◯◯ Low

CI, confidence interval; MD, mean difference; OR, odds ratio; SMD, standardised mean difference.

#### Additional analyses

Given the substantial heterogeneity observed in several pooled outcomes, sensitivity analyses were additionally performed by sequentially removing individual studies to evaluate the robustness of the pooled results and explore potential sources of heterogeneity (Supplementary Table S2). For efficacy outcomes, the main analysis for clinical response rate was not statistically significant. However, exclusion of Cesarani et al. (22) reduced heterogeneity from 71 to 3% and resulted in a statistically significant pooled estimate (OR = 3.00, 95% CI: 1.61 to 5.62). For the 11-point box scale, the main analysis showed a statistically significant effect, and removal of Napryeyenko et al. (24) substantially reduced heterogeneity from 94 to 0% while maintaining a consistent direction of effect (MD = −0.44, 95% CI: −0.60 to −0.28). However, removal of Ihl et al. (25) resulted in a non-significant pooled estimate (MD = −0.90, 95% CI: −1.88 to 0.08), suggesting that the pooled estimate for this outcome showed some sensitivity to the inclusion of individual studies. For safety outcomes, the main analysis for headache was not statistically significant. However, exclusion of Ihl et al. (25) alone reduced heterogeneity from 50 to 0% and showed a statistically significant result (OR = 0.54, 95% CI: 0.39 to 0.76). Similarly, the main analysis for hypertension was not statistically significant, and exclusion of Ihl et al. (25) resulted in a pooled estimate of OR = 0.55 (95% CI, 0.31 to 0.99). In contrast, respiratory tract infection, diarrhea, and tinnitus showed no meaningful changes in effect size or statistical significance across sensitivity analyses, remaining consistent with the main analysis results.

Publication bias could not be formally assessed, as the number of studies contributing to each outcome ranged from two to six, which did not meet the minimum threshold of ten studies recommended for funnel plot assessment.

**Figure 6:**
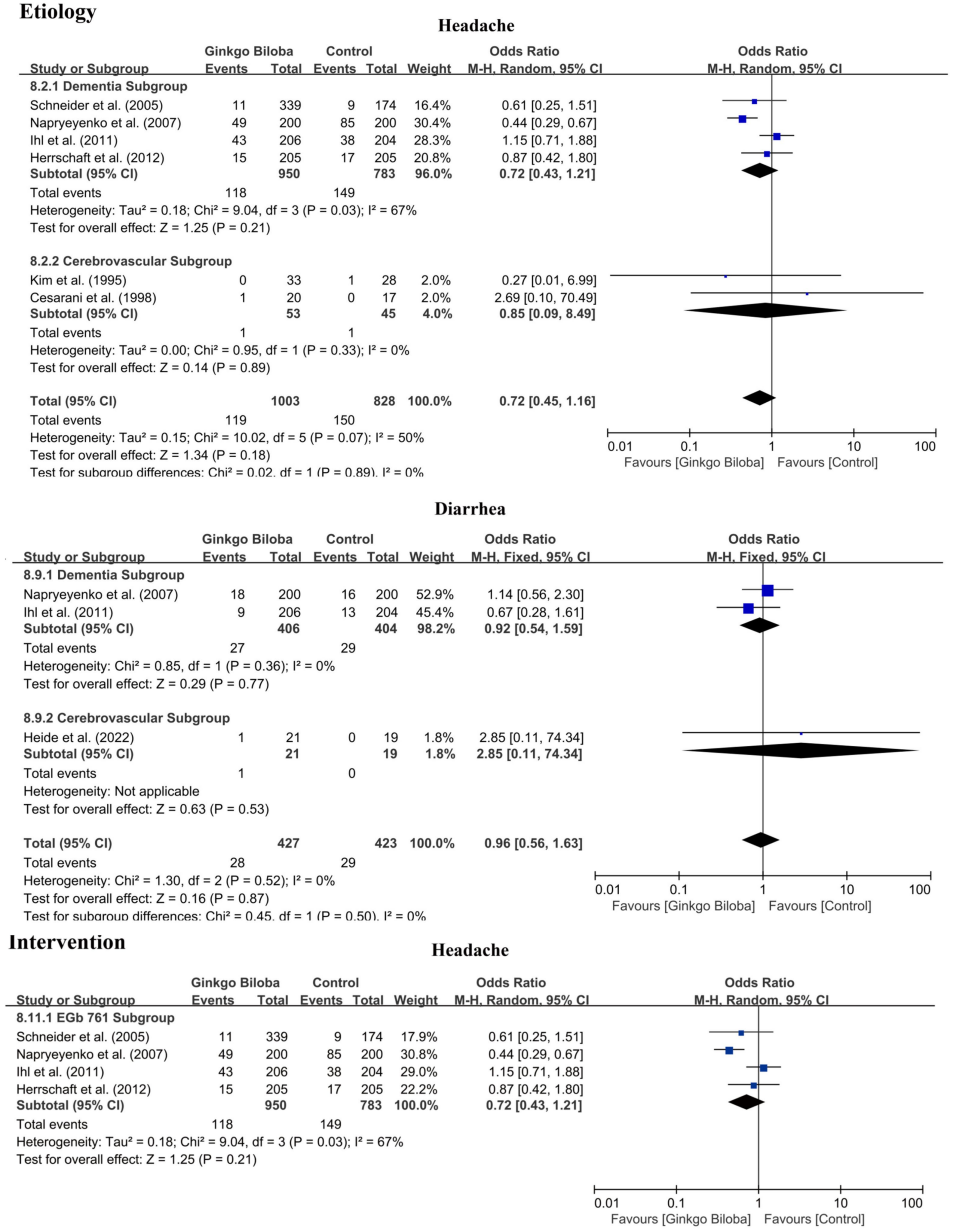
Subgroup meta-analysis by etiology and intervention.

Prespecified subgroup analyses according to underlying etiology and intervention type were performed when at least two studies were available for pooling (Figure 6). For several outcomes, subgroup analyses were not feasible because only one study contributed data within a subgroup. In other cases, subgroup results were identical to the main analysis because the same studies constituted most of the pooled dataset.

In dementia subgroup, dizziness was assessed using the 11-point box scale in three dementia trials. *Ginkgo biloba* extract significantly reduced dizziness scores compared with control (MD = −0.76, 95% CI −1.35 to −0.18), although heterogeneity was considerable (*I*^2^ = 94%). In the same subgroup, pooled safety analyses showed no statistically significant difference in the risk of headache (OR = 0.72, 95% CI: 0.43 to 1.21) or diarrhea (OR = 0.92, 95% CI: 0.54 to 1.59) between the *Ginkgo biloba* and control groups.

In cerebrovascular subgroup, ginkgo biloba extract also significantly reduced VAS compared with control (SMD = −0.38, 95% CI −0.58 to −0.19), with no observed heterogeneity (*I*^2^ = 0%). For safety outcomes in this subgroup, no statistically significant difference in headache risk was observed between groups (OR = 0.85, 95% CI: 0.09 to 8.49).

Subgroup analyses according to intervention type were also performed for studies (22–26, 28) using EGb 761®. Within the EGb 761 subgroup, no statistically significant difference in headache risk was observed between groups (OR = 0.72, 95% CI: 0.43 to 1.21). Regarding mode of administration, only one of the nine included studies administered ginkgo biloba as an add-on to standard care. Given that a meaningful subgroup comparison was not feasible with a single study, the planned subgroup analysis was not performed.

## Discussion

This systematic review and meta-analysis synthesized evidence from nine RCTs to evaluate the efficacy and safety of *Ginkgo biloba* extract in patients with dizziness or vertigo associated with central neurological disorders. The principal findings can be summarized as follows. First, *Ginkgo biloba* significantly reduced dizziness severity, as measured by the 11-point box scale and the VAS, with moderate to low certainty of evidence. Second, *Ginkgo biloba* conferred clinically meaningful and statistically robust improvements in activities of daily living (ADL-IS) and quality of life (DEMQOL-Proxy), supported by moderate-certainty evidence. These outcomes are notable because ADL-IS and DEMQOL-Proxy reflect functional status and quality of life in patients with dementia disorders. Third, the safety profile of *Ginkgo biloba* was favorable; the drug was associated with significantly lower risks of angina pectoris and tinnitus compared to controls, and no significant between-group differences were identified for other adverse events, including headache, gastrointestinal symptoms, or respiratory tract infections. These findings suggest that *Ginkgo biloba* may provide clinically relevant benefits in patients with dizziness or vertigo associated with central neurological disorders, with a favorable safety profile and no significant increase in adverse events.

Sensitivity analyses provided additional insight into the robustness of pooled estimates. For clinical response rate, the non-significant finding in the main analysis was accompanied by substantial heterogeneity. Exclusion of Cesarani et al. (22) yielded a statistically significant pooled estimate with markedly reduced heterogeneity, suggesting that the overall result was sensitive to the inclusion of this study. This may partly reflect differences in study design and population characteristics compared to the other trials; however, given the small number of included studies, this finding should be interpreted with caution. For the 11-point box scale, the direction of effect consistently favored ginkgo biloba across sensitivity analyses. However, exclusion of one study (25) resulted in a non-significant pooled estimate, suggesting that the overall result for this outcome was dependent on the inclusion of individual studies and should be interpreted with caution. Regarding safety, sensitivity analyses did not meaningfully change the pooled estimates for most adverse events. For outcomes that were not statistically significant in the main analysis, such as headache and hypertension, exclusion of one study (25) shifted the estimates toward statistical significance in some cases, while the direction of effect consistently favored ginkgo biloba. Other adverse events showed no meaningful changes across sensitivity analyses and no indication of increased risk associated with ginkgo biloba. Overall, these findings suggest that ginkgo biloba extract was generally well tolerated in the populations studied in whom dizziness, vertigo-, balance-related symptoms, or related functional outcomes were assessed. The inhibition of PAF by ginkgolides may enhance vertebrobasilar circulation, thereby addressing ischemic mechanisms commonly implicated in dizziness-related symptoms in central neurological disorders. Concurrently, the antioxidant and anti-inflammatory properties of flavonoid glycosides may reduce neuroinflammation within central vestibular pathways, facilitating vestibular compensation. These mechanisms align with the pathophysiological processes underlying central vertigo, supporting the biological plausibility of the observed clinical effects.

These mechanisms are supported by individual trials demonstrating improvements in dizziness severity and functional outcomes across various central vertigo etiologies, including cerebrovascular and dementia conditions. Rina et al. demonstrated significant improvements in dizziness scores, DHI, and VAS in dizzy patients with cerebral arteriosclerosis (27). Heide et al., the only trial exclusively enrolling patients with vertebrobasilar transient ischemic attack or infarction, further supported efficacy using objective electronystagmography and vertigo scoring (28). In patients with central vertigo of cerebrovascular origin, one study demonstrated that *Ginkgo biloba* showed efficacy comparable to betahistine, while another reported that its addition to betahistine resulted in greater symptom improvement than betahistine alone, suggesting a potential additive effect (22, 29). Furthermore, *Ginkgo biloba* was associated with improvements in saccadic eye movements and visuovestibular reflexes, indicating enhanced central vestibular compensation, whereas its effects on smooth pursuit were limited (22). The observed improvements in ADL-IS and DEMQOL-Proxy underscore the functional relevance of these findings, particularly in patients with coexisting dementia disorders. Unlike peripheral vestibular disorders, central vertigo associated with dementia or vascular cognitive impairment is often chronic and functionally disabling. These results suggest potential benefits not only for vestibular symptoms but also for overall daily functioning and quality of life. Our findings are consistent with previous studies. By synthesizing evidence across etiologically diverse populations with central vertigo, this meta-analysis provides more comprehensive estimates than individual studies. The reduced incidence of tinnitus observed in patients treated with *Ginkgo biloba* may also be clinically relevant, as tinnitus frequently coexists with central vertigo of vascular origin and may reflect improved cochleovestibular microcirculation.

Several limitations of this review should be acknowledged. First, the number of eligible RCTs was relatively small (*n* = 9), and many outcomes were derived from only a limited subset of studies (22–26). Second, the included trials were clinically heterogeneous with respect to underlying etiology, *Ginkgo biloba* formulation, comparator type, and treatment duration. To address this heterogeneity, subgroup analyses according to underlying etiology and intervention type were conducted. However, subgroup analyses could only be performed for a limited number of outcomes, and several planned subgroup analyses were not feasible because only a single study contributed data within a subgroup. Therefore, the ability to fully explore potential sources of heterogeneity was limited. Third, the overall risk of bias was moderate to high, with several studies exhibiting concerns related to blinding and selective outcome reporting. Fourth, commonly used outcome measures such as the 11-point box scale and VAS primarily reflect subjective symptom severity and do not provide objective assessments of central vestibular function, although a limited number of studies incorporated objective oculomotor and vestibular assessments (30). Fifth, as *Ginkgo biloba* has been studied in several non-English-language settings, including Chinese, German, and French literature, eligible studies may have been missed due to language restrictions. In this review, we limited eligible publications to English and Korean because these were the languages in which the review team could reliably assess eligibility, extract data, evaluate risk of bias, and interpret clinical and methodological details. Therefore, language restriction may have introduced selection bias and may have affected the completeness of the evidence base. Finally, publication bias could not be formally assessed due to the limited number of included studies.

Despite these limitations, the findings provide clinically relevant insights. *Ginkgo biloba* extract may represent a therapeutic option for patients with central vertigo, particularly those with underlying cerebrovascular or neurodegenerative conditions in whom vestibular suppressants are limited by adverse effects such as sedation or cognitive impairment. The alignment between its pharmacological mechanisms and the pathophysiology of central vertigo supports its potential clinical relevance (9–12).

Future research should focus on well-designed, double-blind randomized controlled trials enrolling patients with clearly defined central vertigo, ideally confirmed by neuroimaging or vestibular testing. Standardized outcome measures specific to central vestibular dysfunction should be employed, and long-term studies are also warranted to determine whether *Ginkgo biloba* extract can influence disease progression or recurrence of vertigo symptoms.

## Conclusion

This systematic review and meta-analysis of nine randomized controlled trials involving 2,394 participants suggests that *Ginkgo biloba* extract may reduce dizziness or vertigo severity across diverse central neurological etiologies, including cerebrovascular disease, vertebrobasilar ischemia, and neurodegenerative disorders (21–29). Significant improvements were observed in vertigo severity, activities of daily living, and quality of life, with a favorable safety profile (25, 26). The safety profile of *Ginkgo biloba* extract was favorable, with significantly reduced risks of angina pectoris and tinnitus without a significant increase in other adverse events such as headache, gastrointestinal events, or respiratory tract infections. The pharmacological properties of EGb 761®, including enhancement of cerebral microcirculation, antioxidant and anti-inflammatory effects, and modulation of neurotransmitter systems, support the biological plausibility of these findings (9–12). Given that central vertigo represents a broad clinical construct encompassing diverse central nervous system pathologies, these findings should be interpreted within this context.

Collectively, these results support the potential role of *Ginkgo biloba* extract as a therapeutic option for vertigo, particularly in patients with cerebrovascular or dementia conditions. However, further high-quality, well-designed randomized controlled trials with clearly defined patient populations are required to confirm these findings and to establish its role in clinical practice.

## Data Availability

The original contributions presented in the study are included in the article/Supplementary material, further inquiries can be directed to the corresponding authors.

## References

[1] Neuhauser H. The epidemiology of dizziness and vertigo. Handb Clin Neurol. (2016) 137:67–82. doi: 10.1016/B978-0-444-63437-5.00005-4, 27638063

[2] Neuhauser H. Epidemiology of dizziness and vertigo. Nervenarzt. (2009) 80:887–94. doi: 10.1007/s00115-009-2738-9, 19626307

[3] Strupp M Brandt T. Diagnosis and treatment of vertigo and dizziness. Dtsch Arztebl Int. (2008) 105:173–80. doi: 10.3238/arztebl.2008.0173, 19629221 PMC2696792

[4] Kerber KA Meurer WJ West BT Mark FA. Dizziness presentations in US emergency departments, 1995–2004. Acad Emerg Med. (2008) 15:744–50. doi: 10.1111/j.1553-2712.2008.00189.x, 18638027

[5] Brandt T Dieterich M. The dizzy patient: don't forget disorders of the central vestibular system. Nat Rev Neurol. (2017) 13:352–62. doi: 10.1038/nrneurol.2017.58, 28429801

[6] Kim JS Lee H. Inner ear Dysfunction due to Vertebrobasilar Ischemic Stroke. Semin Neurol. (2009) 29:534–40. doi: 10.1055/s-0029-1241037 19834865

[7] Herdman SJ. Vestibular rehabilitation. Curr Opin Neurol. (2013) 26:96–101. doi: 10.1097/WCO.0b013e32835c5ec4, 23241567

[8] Murdin L Hussain K Schilder AG. Betahistine for symptoms of vertigo. Cochrane Database Syst Rev. (2016) 2020:CD010696. doi: 10.1002/14651858.CD010696.pub2, 27327415 PMC7388750

[9] Braquet P. Ginkgolides: potent platelet activating factor antagonists isolated from *Ginkgo biloba* L: chemistry, pharmacology and clinical applications. Drugs of The Future. (1987) 12:643–99. doi: 10.1358/DOF.1987.012.07.77903

[10] Luo Y Smith JV Paramasivam V Burdick A Curry KJ Buford JP . Inhibition of amyloid-β aggregation and caspase-3 activation by the *Ginkgo biloba* extract EGb761. Proc Natl Acad Sci. (2002) 99:12197–202. doi: 10.1073/pnas.182425199, 12213959 PMC129421

[11] Ahlemeyer B Krieglstein J. Neuroprotective effects of *Ginkgo biloba* extract. Cell Mol Life Sci. (2003) 60:1779–92. doi: 10.1007/s00018-003-3080-1, 14523543 PMC11146048

[12] DeFeudis F Drieu K. *Ginkgo biloba* extract (EGb 761) and CNS functions basic studies and clinical applications. Curr Drug Targets. (2000) 1:25–58. doi: 10.2174/1389450003349380, 11475535

[13] Sokolova L Hoerr R Mishchenko T. Treatment of Vertigo: a randomized, double-blind trial comparing efficacy and safety of *Ginkgo biloba* extract EGb 761 and Betahistine. Int J Otolaryngol. (2014) 2014:682439. doi: 10.1155/2014/682439, 25057270 PMC4099171

[14] Page MJ McKenzie JE Bossuyt PM Boutron I Hoffmann TC Mulrow CD . The PRISMA 2020 statement: an updated guideline for reporting systematic reviews. BMJ. (2021):372. doi: 10.1136/bmj.n71, 33782057 PMC8005924

[15] Isah T. Rethinking *Ginkgo biloba* L.: medicinal uses and conservation. Pharmacogn Rev. (2015) 9:140. doi: 10.4103/0973-7847.162137, 26392712 PMC4557237

[16] Sterne JA Savović J Page MJ Elbers RG Blencowe NS Boutron I . RoB 2: a revised tool for assessing risk of bias in randomised trials. BMJ. (2019) 366:l4898. doi: 10.1136/bmj.l4898 31462531

[17] DerSimonian R Laird N. Meta-analysis in clinical trials. Control Clin Trials. (1986) 7:177–88. doi: 10.1016/0197-2456(86)90046-2, 3802833

[18] Chandler J Cumpston M Li T Page MJ Welch V. Cochrane Handbook for Systematic Reviews of Interventions. Hoboken: Wiley (2019).

[19] Guyatt GH Oxman AD Vist GE Kunz R Falck-Ytter Y Alonso-Coello P . GRADE: an emerging consensus on rating quality of evidence and strength of recommendations. BMJ. (2008) 336:924–6. doi: 10.1136/bmj.39489.470347.AD, 18436948 PMC2335261

[20] Balshem H Helfand M Schünemann HJ Oxman AD Kunz R Brozek J . GRADE guidelines: 3. Rating the quality of evidence. J Clin Epidemiol. (2011) 64:401–6. doi: 10.1016/j.jclinepi.2010.07.015, 21208779

[21] Kim J-S Lee S-H Kim J-M Lee K-W. Studies on the efficacy and safety of *Ginkgo Biloba* extract (Tanamin^R^) in the dizziness of central origin. J Korean Soc Clin Pharmacol Ther. (1995) 3:187–97. doi: 10.12793/jkscpt.1995.3.2.187

[22] Cesarani A Meloni F Alpini D Barozzi S Verderio L Boscani P. *Ginkgo biloba* (EGb 761) in the treatment of equilibrium disorders. Adv Ther. (1998) 15:291–304. 10345150

[23] Schneider LS DeKosky ST Farlow MR Tariot PN Hoerr R Kieser M. A randomized, double-blind, placebo-controlled trial of two doses of *Ginkgo biloba* extract in dementia of the Alzheimer's type. Curr Alzheimer Res. (2005) 2:541–51. doi: 10.2174/156720505774932287, 16375657

[24] Napryeyenko O Borzenko I. *Ginkgo biloba* special extract in dementia with neuropsychiatric features. Arzneimittelforschung. (2007) 57:4–11. doi: 10.1055/s-0031-1296579 17341003

[25] Ihl R Bachinskaya N Korczyn AD Vakhapova V Tribanek M Hoerr R . Efficacy and safety of a once-daily formulation of *Ginkgo biloba* extract EGb 761 in dementia with neuropsychiatric features: a randomized controlled trial. Int J Geriatr Psychiatry. (2011) 26:1186–94. doi: 10.1002/gps.2662, 21140383

[26] Herrschaft H Nacu A Likhachev S Sholomov I Hoerr R Schlaefke S. *Ginkgo biloba* extract EGb 761® in dementia with neuropsychiatric features: a randomised, placebo-controlled trial to confirm the efficacy and safety of a daily dose of 240 mg. J Psychiatr Res. (2012) 46:716–23. doi: 10.1016/j.jpsychires.2012.03.003, 22459264

[27] Rina S Lu T Yawei D Shengxian W Huawei S Hongxin Z . Effectiveness and safety of *Ginkgo biloba* extract (GBE50) in the treatment of dizziness caused by cerebral arteriosclerosis: a multi-center, double-blind, randomized controlled trial. J Tradit Chin Med. (2021) 42:83–9. doi: 10.19852/j.cnki.jtcm.20211214.001 PMC10164630 35294126

[28] Heide W Adlung B Körtke C Hoerr R. *Ginkgo biloba* extract EGb 761® improves central vestibular vertigo in patients undergoing vestibular exercises: a randomised placebo-controlled trial. Neuroscience and Medicine. (2022) 13:91–102. doi: 10.4236/nm.2022.133008

[29] Li C Cao L. Evaluating the effectiveness of *Ginkgo Biloba* extract in alleviating dizziness and improving nutritional status in cerebral arteriosclerosis patients. Current topics in nutraceutical. Research. (2024) 22:174–80. doi: 10.37290/ctnr2641-452X.22:174-180, 42238678

[30] Sterne JA Sutton AJ Ioannidis JP Terrin N Jones DR Lau J . Recommendations for examining and interpreting funnel plot asymmetry in meta-analyses of randomised controlled trials. BMJ. (2011) 343:d4002. doi: 10.1136/bmj.d4002, 21784880

