# Supplementary material for: Ginkgo biloba extract for dizziness-related symptoms in central neurological disorders: a systematic review and meta-analysis
Source: Front Neurol. 2026 Jun 18;17:1860538. doi: 10.3389/fneur.2026.1860538 (PMC13322805; doi:10.3389/fneur.2026.1860538)
Supplement: Supplementary file 1 [file Table_1.DOCX]

**Supplementary Table 1.** Search strategy for each database

| **Database** | **No.** | **Search Query** | **Results** |
| --- | --- | --- | --- |
| **PubMed** | #1 | "Vertigo"[Mesh] | 12,906 |
|  | #2 | "Vestibular Diseases"[Mesh:NoExp] | 5,395 |
|  | #3 | "Dizziness"[Mesh] | 7,119 |
|  | #4 | "Vertebrobasilar Insufficiency"[Mesh] | 5,696 |
|  | #5 | "Postural Balance"[Mesh] | 31,930 |
|  | #6 | vertigo*[TW] OR vertiginous[TW] OR "vestibular disease*"[TW] OR spinning[TW] OR dizziness[TW] OR lightheadedness[TW] OR light-headedness[TW] OR giddiness[TW] | 67,057 |
|  | #7 | "postural balanc*"[TW] OR "posture balanc*"[TW] OR "posture equilibrium*"[TW] OR "postural equilibrium*"[TW] OR "postural control*"[TW] OR "posture control*"[TW] OR "postural instabilit*"[TW] OR disequilibrium*[TW] OR unsteadiness[TW] OR "balance disorder*"[TW] | 77,958 |
|  | #8 | #1 OR #2 OR #3 OR #4 OR #5 OR #6 OR #7 | 146,603 |
|  | #9 | "Ginkgo biloba"[Mesh] | 3,784 |
|  | #10 | "Ginkgo Extract"[Mesh] | 89 |
|  | #11 | "Ginkgo biloba extract"[Supplementary Concept] | 1,251 |
|  | #12 | "Ginkgo biloba extract 50"[Supplementary Concept] | 8 |
|  | #13 | ginkgo*[TW] OR gingko*[TW] OR ginko*[TW] OR biloba*[TW] OR maidenhair[TW] OR tebofortran[TW] OR tebokan[TW] OR tebonin[TW] OR egb761[TW] OR egb-761[TW] OR rokan[TW] OR tanakan[TW] OR gbe761[TW] OR gbe-761[TW] | 9,342 |
|  | #14 | egb50[TW] OR egb-50[TW] OR gbe50[TW] OR gbe-50[TW] | 51 |
|  | #15 | ginkobene[TW] OR ginkor[TW] OR kaveri[TW] OR li1370[TW] OR li-1370[TW] OR superginko[TW] OR tanakene[TW] OR bilobil[TW] | 90 |
|  | #16 | #9 OR #10 OR #11 OR #12 OR #13 OR #14 OR #15 | 9,388 |
|  | #17 | #8 AND #16 | 103 |
|  | #18 | #17 NOT (animals[Mesh:noexp] NOT (animals[Mesh:noexp] AND humans[Mesh])) | 95 |
|  | #19 | #18 AND (("Controlled Clinical Trials as Topic"[Mesh] OR "Randomized Controlled Trials as Topic"[Mesh] OR Randomized Controlled Trial[PT] OR Controlled Clinical Trial[PT] OR Multicenter Study[PT] OR "Clinical Trials as topic"[Mesh] OR Random*[TW] OR randomized[TW] OR randomised[TW] OR ((Double*[TW] OR single*[TW] OR treb*[TW] OR tripl*[TW]) AND (Blind*[TW] OR mask*[TW])) OR controlled-clinical-trial*[TW] OR controlled-trial*[TW] OR placebo*[TW] OR randomly*[TW]) NOT (Case Reports[PT] OR Letter[PT] OR meta-analys*[TW] OR metaanalys*[TW] OR "Meta-Analysis" [PT] OR case-report*[TW] OR Letter*[TI] OR “Systematic Review”[PT] OR Systematic-Review*[TI])) | 34 |
|  | #20 | #19 AND (english[la] OR korean[la]) AND (1974:2025/11/30[dp]) | **18** |
| **EMBASE** | #1 | 'vertigo'/exp | 62,667 |
|  | #2 | 'vestibular disorder'/de | 12,535 |
|  | #3 | 'balance disorder'/exp | 190,993 |
|  | #4 | 'dizziness'/exp | 124,374 |
|  | #5 | 'vertebrobasilar insufficiency'/exp | 3,615 |
|  | #6 | 'body equilibrium'/exp | 28,401 |
|  | #7 | vertigo*:ab,ti,kw OR vertiginous:ab,ti,kw OR "vestibular disease*":ab,ti,kw OR spinning:ab,ti,kw OR dizziness:ab,ti,kw OR lightheadedness:ab,ti,kw OR light-headedness:ab,ti,kw OR giddiness:ab,ti,kw | 89,323 |
|  | #8 | (postur* NEXT balanc*):ab,ti,kw OR (postur* NEXT equilibrium*):ab,ti,kw OR (postur* NEXT control*):ab,ti,kw OR (postural NEXT instabilit*):ab,ti,kw OR disequilibrium*:ab,ti,kw OR unsteadiness:ab,ti,kw OR (balance NEXT disorder*):ab,ti,kw | 38,319 |
|  | #9 | #1 OR #2 OR #3 OR #4 OR #5 OR #6 OR #7 OR #8 | 384,632 |
|  | #10 | 'Ginkgo'/exp | 6,480 |
|  | #11 | 'Ginkgo biloba'/exp | 6,197 |
|  | #12 | 'Ginkgo biloba extract'/exp | 8,585 |
|  | #13 | ginkgo*:ab,ti,kw OR gingko*:ab,ti,kw OR ginko*:ab,ti,kw OR biloba*:ab,ti,kw OR maidenhair:ab,ti,kw OR tebofortran:ab,ti,kw OR tebokan:ab,ti,kw OR tebonin:ab,ti,kw OR egb761:ab,ti,kw OR egb-761:ab,ti,kw OR rokan:ab,ti,kw OR tanakan:ab,ti,kw OR gbe761:ab,ti,kw OR gbe-761:ab,ti,kw | 12,347 |
|  | #14 | egb50:ab,ti,kw OR egb-50:ab,ti,kw OR gbe50:ab,ti,kw OR gbe-50:ab,ti,kw | 80 |
|  | #15 | ginkobene:ab,ti,kw OR ginkor:ab,ti,kw OR kaveri:ab,ti,kw OR li1370:ab,ti,kw OR li-1370:ab,ti,kw OR superginko:ab,ti,kw OR tanakene:ab,ti,kw OR bilobil:ab,ti,kw | 101 |
|  | #16 | #10 OR #11 OR #12 OR #13 OR #14 OR #15 | 17,960 |
|  | #17 | #9 AND #16 | 843 |
|  | #18 | #17 NOT ('animal'/de NOT ('animal'/de AND 'human'/exp)) | 843 |
|  | #19 | #18 AND (('randomized controlled trial'/exp OR [randomized controlled trial]/lim OR 'controlled clinical trial'/de OR 'multicenter study'/exp OR 'randomization'/exp OR (Random* OR randomized OR randomised OR controlled-clinical-trial* OR controlled-trial* OR placebo* OR randomly*):ab,ti,kw OR ((Double* OR single* OR treb* OR tripl*) NEAR/3 (Blind* OR mask*)):ab,ti,kw) NOT ([conference abstract]/lim OR [conference paper]/lim OR [conference review]/lim OR [data papers]/lim OR [editorial]/lim OR [erratum]/lim OR [letter]/lim OR [note]/lim OR [review]/lim)) | 129 |
|  | #20 | #19 AND ([english]/lim OR [korean]/lim) AND [01-01-1900]/sd NOT [01-12-2025]/sd | **109** |
| **Cochrane CENTRAL** | #1 | [mh "Vertigo"] | 796 |
|  | #2 | [mh ^"Vestibular Diseases"] | 284 |
|  | #3 | [mh "Dizziness"] | 1,075 |
|  | #4 | [mh "Vertebrobasilar Insufficiency"] | 100 |
|  | #5 | [mh "Postural Balance"] | 4,706 |
|  | #6 | vertigo*:ab,ti,kw OR vertiginous:ab,ti,kw OR (vestibular NEXT disease*):ab,ti,kw OR spinning:ab,ti,kw OR dizziness:ab,ti,kw OR lightheadedness:ab,ti,kw OR light-headedness:ab,ti,kw OR giddiness:ab,ti,kw | 21,673 |
|  | #7 | (postur* NEXT balanc*):ab,ti,kw OR (postur* NEXT equilibrium*):ab,ti,kw OR (postur* NEXT control*):ab,ti,kw OR (postural NEXT instabilit*):ab,ti,kw OR disequilibrium*:ab,ti,kw OR unsteadiness:ab,ti,kw OR (balance NEXT disorder*):ab,ti,kw | 8,455 |
|  | #8 | #1 OR #2 OR #3 OR #4 OR #5 OR #6 OR #7 | 29,694 |
|  | #9 | [mh "Ginkgo biloba"] | 384 |
|  | #10 | [mh "Ginkgo Extract"] | 12 |
|  | #11 | ginkgo*:ab,ti,kw OR gingko*:ab,ti,kw OR ginko*:ab,ti,kw OR biloba*:ab,ti,kw OR maidenhair:ab,ti,kw OR tebofortran:ab,ti,kw OR tebokan:ab,ti,kw OR tebonin:ab,ti,kw OR egb761:ab,ti,kw OR egb-761:ab,ti,kw OR rokan:ab,ti,kw OR tanakan:ab,ti,kw OR gbe761:ab,ti,kw OR gbe-761:ab,ti,kw | 1,465 |
|  | #12 | egb50:ab,ti,kw OR egb-50:ab,ti,kw OR gbe50:ab,ti,kw OR gbe-50:ab,ti,kw | 1 |
|  | #13 | ginkobene:ab,ti,kw OR ginkor:ab,ti,kw OR kaveri:ab,ti,kw OR li1370:ab,ti,kw OR li-1370:ab,ti,kw OR superginko:ab,ti,kw OR tanakene:ab,ti,kw OR bilobil:ab,ti,kw | 25 |
|  | #14 | #9 OR #10 OR #11 OR #12 OR #13 | 1,470 |
|  | #15 | #8 AND #14 | 81 |
|  | #16 | #15 NOT ([mh ^"animals"] NOT ([mh ^"animals"] AND [mh "humans"])) | 81 |
|  | #17 | #16 with Publication Year from 1900 to 2025, Language English/Korean, in Trials(CENTRAL) | 60 |
|  | #18 | #17 - Published articles | **48** |
| **CINAHL** | #1 | MH "Vertigo+" OR MH "Vestibular Diseases" OR MH "Dizziness" OR MH "Balance, Postural+" OR vertigo* OR vertiginous OR "vestibular disease*" OR spinning OR dizziness OR lightheadedness OR light-headedness OR giddiness OR "postural balanc*" OR "posture balanc*" OR "posture equilibrium*" OR "postural equilibrium*" OR "postural control*" OR "posture control*" OR "postural instabilit*" OR disequilibrium* OR unsteadiness OR "balance disorder*" |  |
|  | #2 | MH "Ginkgo Biloba" OR ginkgo* OR gingko* OR ginko* OR biloba* OR maidenhair OR tebofortran OR tebokan OR tebonin OR egb761 OR egb-761 OR rokan OR tanakan OR gbe761 OR gbe-761 OR egb50 OR egb-50 OR gbe50 OR gbe-50 OR ginkobene OR ginkor OR kaveri OR li1370 OR li-1370 OR superginko OR tanakene OR bilobil |  |
|  | #3 | #1 AND #2 | 32 |
|  | #4 | Filters : 1900.01.01.-2025.11.30, Language English/Korean, Academic Journals | **27** |
| **Web of Science** | #1 | TS=(vertigo* OR vertiginous OR "vestibular disease*" OR spinning OR dizziness OR lightheadedness OR light-headedness OR giddiness OR "postural balanc*" OR "posture balanc*" OR "posture equilibrium*" OR "postural equilibrium*" OR "postural control*" OR "posture control*" OR "postural instabilit*" OR disequilibrium* OR unsteadiness OR "balance disorder*") | 771,489 |
|  | #2 | TS=(ginkgo* OR gingko* OR ginko* OR biloba* OR maidenhair OR tebofortran OR tebokan OR tebonin OR egb761 OR egb-761 OR rokan OR tanakan OR gbe761 OR gbe-761 OR egb50 OR egb-50 OR gbe50 OR gbe-50 OR ginkobene OR ginkor OR kaveri OR li1370 OR li-1370 OR superginko OR tanakene OR bilobil) | 13,341 |
|  | #3 | #1 AND #2 | 144 |
|  | #4 | #3 AND TS=(random* OR randomized OR randomised OR ((double* OR single* OR treb* OR tripl*) AND (blind* OR mask*)) OR controlled-clinical-trial* OR controlled-trial* OR placebo* OR randomly*) | 54 |
|  | #5 | #4 AND DOP=(1990-01-01/2025-11-30) AND LA=(English OR Korean) | **50** |
| **KoreaMed** | #1 | (vertigo[MH] OR "Vestibular Diseases"[MH] OR dizziness[MH] OR "Vertebrobasilar Insufficiency"[MH] OR "Postural Balance"[MH] OR vertigo*[ALL] OR vertiginous[ALL] OR "vestibular disease*"[ALL] OR spinning[ALL] OR dizziness[ALL] OR lightheadedness[ALL] OR light-headedness[ALL] OR giddiness[ALL] OR "postural balanc*"[ALL] OR "posture balanc*"[ALL] OR "posture equilibrium*"[ALL] OR "postural equilibrium*"[ALL] OR "postural control*"[ALL] OR "posture control*"[ALL] OR "postural instabilit*"[ALL] OR disequilibrium*[ALL] OR unsteadiness[ALL] OR "balance disorder*"[ALL]) AND ("ginkgo biloba"[MH] OR "ginkgo extract"[MH] OR ginkgo*[ALL] OR gingko*[ALL] OR ginko*[ALL] OR biloba*[ALL] OR maidenhair[ALL] OR tebofortran[ALL] OR tebokan[ALL] OR tebonin[ALL] OR egb761[ALL] OR egb-761[ALL] OR rokan[ALL] OR tanakan[ALL] OR gbe761[ALL] OR gbe-761[ALL] OR egb50[ALL] OR egb-50[ALL] OR gbe50[ALL] OR gbe-50[ALL] OR ginkobene[ALL] OR ginkor[ALL] OR kaveri[ALL] OR li1370[ALL] OR li-1370[ALL] OR superginko[ALL] OR tanakene[ALL] OR bilobil[ALL]) AND 1900:2025[DPY] | 0 |
| **RISS** | #1 | vertigo*\|vertiginous\|"vestibular disease*"\|spinning\|dizziness\|lightheadedness\|light-headedness\|giddiness\|"postural balanc*"\|"posture balanc*"\|"posture equilibrium*"\|"postural equilibrium*"\|"postural control*"\|"posture control*"\|"postural instabilit*"\|disequilibrium*\|unsteadiness\|"balance disorder*” |  |
|  | #2 | ginkgo*\|gingko*\|ginko*\|biloba*\|maidenhair\|tebofortran\|tebokan\|tebonin\|egb761\|egb-761\|rokan\|tanakan\|gbe761\|gbe-761\|egb50\|egb-50\|gbe50\|gbe-50\|ginkobene\|ginkor\|kaveri\|li1370\|li-1370\|superginko\|tanakene\|bilobil |  |
|  | #3 | #1 AND #2 | **4** |
| **ScienceON** | #1 | vertigo*\|vertiginous\|"vestibular disease*"\|spinning\|dizziness\|lightheadedness\|light-headedness\|giddiness\|"postural balanc*"\|"posture balanc*"\|"posture equilibrium*"\|"postural equilibrium*"\|"postural control*"\|"posture control*"\|"postural instabilit*"\|disequilibrium*\|unsteadiness\|"balance disorder*” |  |
|  | #2 | ginkgo*\|gingko*\|ginko*\|biloba*\|maidenhair\|tebofortran\|tebokan\|tebonin\|egb761\|egb-761\|rokan\|tanakan\|gbe761\|gbe-761\|egb50\|egb-50\|gbe50\|gbe-50\|ginkobene\|ginkor\|kaveri\|li1370\|li-1370\|superginko\|tanakene\|bilobil |  |
|  | #3 | #1 AND #2 | **4** |
| **KMbase** | #1 | ((vertigo*\|keyword) OR (vertiginous\|keyword) OR ("vestibular disease*"\|keyword) OR (spinning\|keyword) OR (dizziness\|keyword) OR (lightheadedness\|keyword) OR (light-headedness\|keyword) OR (giddiness\|keyword) OR ("postural balanc*"\|keyword) OR ("posture balanc*"\|keyword) OR ("posture equilibrium*"\|keyword) OR ("postural equilibrium*"\|keyword) OR ("postural control*"\|keyword) OR ("posture control*"\|keyword) OR ("postural instabilit*"\|keyword) OR (disequilibrium*\|keyword) OR (unsteadiness\|keyword) OR ("balance disorder*”\|keyword)) AND ((ginkgo*\|keyword) OR (gingko*\|keyword) OR (ginko*\|keyword) OR (biloba*\|keyword) OR (maidenhair\|keyword) OR (tebofortran\|keyword) OR (tebokan\|keyword) OR (tebonin\|keyword) OR (egb761\|keyword) OR (egb-761\|keyword)) | **0** |
