# Supplementary material for: Ginkgo biloba extract for dizziness-related symptoms in central neurological disorders: a systematic review and meta-analysis
Source: Front Neurol. 2026 Jun 18;17:1860538. doi: 10.3389/fneur.2026.1860538 (PMC13322805; doi:10.3389/fneur.2026.1860538)
Supplement: Supplementary file 2 [file Table_2.DOCX]

**Supplementary Table 2.** Sensitivity analysis of the pooled estimate of efficacy and safety of ginkgo biloba

| Outcome | Leave-one-out * | Pooled estimate [95% CI] | Heterogeneity | |
| --- | --- | --- | --- | --- |
|  |  |  | I² (%) | p |
| Efficacy | | | | |
| Clinical response rate | All studies | 1.82 [0.42, 7.9] | 71 | 0.42 |
|  | Kim et al. (1995) | 0.82 [0.06, 11.79] | 81 | 0.88 |
|  | Cesarani et al. (1998) | 3.00 [1.61, 5.62] | 3 | <0.001 |
|  | Rina et al. (2021) | 1.10 [0.03, 36.99] | 86 | 0.96 |
| 11-point box scale | All studies | -0.76 [-1.35, -0.18] | 94 | 0.01 |
|  | Napryeyenko et al. (2007) | -0.44 [-0.60, -0.28] | 0 | <0.001 |
|  | Ihl et al. (2011) | -0.90 [-1.88, 0.08] | 97 | 0.07 |
|  | Herrschaft et al. (2012) | -0.95 [-1.83, -0.07] | 95 | 0.04 |
| Safety | | | | |
| Headache | All studies | 0.72 [0.45, 1.16] | 50 | 0.18 |
|  | Kim et al. (1995) | 0.74 [0.45, 1.22] | 59 | 0.24 |
|  | Cesarani et al. (1998) | 0.70 [0.43, 1.15] | 57 | 0.16 |
|  | Schneider et al. (2005) | 0.75 [0.42, 1.35] | 60 | 0.34 |
|  | Napryeyenko et al. (2007) | 0.96 [0.67, 1.38] | 0 | 0.84 |
|  | Ihl et al. (2011) | 0.54 [0.39, 0.76] | 0 | <0.001 |
|  | Herrschaft et al. (2012) | 0.69 [0.38, 1.27] | 58 | 0.23 |
| Dizziness | All studies | 0.55 [0.26, 1.14] | 73 | 0.11 |
|  | Schneider et al. (2005) | 0.41 [0.19, 0.89] | 65 | 0.02 |
|  | Napryeyenko et al. (2007) | 0.70 [0.33, 1.50] | 63 | 0.36 |
|  | Ihl et al. (2011) | 0.46 [0.16, 1.33] | 80 | 0.15 |
|  | Herrschaft et al. (2012) | 0.66 [0.29, 1.48] | 77 | 0.31 |
| Respiratory tract infection | All studies | 1.10 [0.76, 1.59] | 0 | 0.62 |
|  | Schneider et al. (2005) | 1.27 [0.80, 2.00] | 0 | 0.31 |
|  | Napryeyenko et al. (2007) | 1.12 [0.75, 1.67] | 0 | 0.59 |
|  | Ihl et al. (2011) | 0.96 [0.61, 1.52] | 0 | 0.87 |
|  | Herrschaft et al. (2012) | 1.07 [0.72, 1.58] | 0 | 0.74 |
| Hypertension/Blood pressure increased | All studies | 0.74 [0.48, 1.13] | 8 | 0.16 |
|  | Napryeyenko et al. (2007) | 0.92 [0.53, 1.59] | 0 | 0.76 |
|  | Ihl et al. (2011) | 0.55 [0.31, 0.99] | 0 | 0.05 |
|  | Herrschaft et al. (2012) | 0.76 [0.48, 1.20] | 51 | 0.24 |
| Diarrhea | All studies | 0.96 [0.56, 1.63] | 0 | 0.87 |
|  | Napryeyenko et al. (2007) | 0.75 [0.33, 1.73] | 0 | 0.50 |
|  | Ihl et al. (2011) | 1.19 [0.60, 2.37] | 0 | 0.61 |
|  | Herrschaft et al. (2012) | 0.92 [0.54, 1.59] | 0 | 0.77 |
| Tinnitus | All studies | 0.37 [0.22, 0.63] | 32 | <0.001 |
|  | Schneider et al. (2005) | 0.31 [0.16, 0.63] | 60 | 0.001 |
|  | Napryeyenko et al. (2007) | 0.31 [0.16, 0.62] | 63 | <0.001 |
|  | Ihl et al. (2011) | 0.49 [0.27, 0.87] | 0 | 0.02 |

* pooled estimate after exclusion of the indicated study
