# Supplementary material for: Ginkgo biloba extract for dizziness-related symptoms in central neurological disorders: a systematic review and meta-analysis
Source: Front Neurol. 2026 Jun 18;17:1860538. doi: 10.3389/fneur.2026.1860538 (PMC13322805; doi:10.3389/fneur.2026.1860538)
Supplement: Supplementary file 3 [file Table_3.DOCX]

**Supplementary Table 3.** Data extraction table of included studies

| **Study** | **Study characteristic** | | **Population** | | **Result** | | | | | |
| --- | --- | --- | --- | --- | --- | --- | --- | --- | --- | --- |
|  | **Year** | **Country** | **T** | **C** | **Outcome** | | **Range of Scores and Scoring Intervention** | **Follow-up period** | **T** | **C** |
|  |  |  | **N** | **N** |  |  |  |  |  |  |
| Kim et al. (1995) ^21^ | 1995 | South Korea | 31 | 26 | Efficacy | Clinical response rate | Range:  1=Remarkably improved  2=Improved  3=Unchanged  4=Aggravated | 6 months | Remarkably improved (n=11)  Improved (n=17)  Unchanged (n=3)  Aggravated (n=0) | Remarkably improved (n=2)  Improved (n=14)  Unchanged (n=9)  Aggravated (n=1) |
|  |  |  | 33 | 28 | Safety | Adverse events | Frequency | 6 months | Abdominal discomfort (n=3)  Nausea/vomiting (n=3)  Headahce (n=1)  Skin rash (n=1) | Abdominal discomfort (n=2)  Nausea/vomiting (n=3)  Headache (n=1)  Skin rash (n=0) |
| Cesarani et al. (1998) ^22^ | 1998 | Italy | 20 | 17 | Efficacy | Rate of improvement of vertigo | Range:  1=Disappeared  2=Improved  3=Unchanged  4=Worsened | 1 month | Disappeared (n=2)  Improved (n=11)  Unchanged (n=5)  Worsened (n=2) | Disappeared (n=0)  Improved (n=11)  Unchanged (n=3)  Worsened (n=3) |
|  |  |  | 19 | 14 |  |  |  | 2 months | Disappeared (n=7)  Improved (n=7)  Unchanged (n=0)  Worsened (n=5) | Disappeared (n=1)  Improved (n=8)  Unchanged (n=1)  Worsened (n=4) |
|  |  |  | 18 | 13 |  |  |  | 3 months | Disappeared (n=8)  Improved (n=4)  Unchanged (n=2)  Worsened (n=4) | Disappeared (n=6)  Improved (n=6)  Unchanged (n=1)  Worsened (n=0) |
|  |  |  | 20 | 17 | Safety | Adverse events | Frequency | Baseline,  1, 2, 3 months | Headache (n=1)  Cyanosis of the nails/lips (n=0)  Gastrointestinal disorders (n=1) | Headache (n=0)  Cyanosis of the nails/lips (n=1)  Gastrointestinal disorders (n=1) |
| Schneider et al. (2005) ^23^ | 2005 | United States | 169/170* | 174 | Efficacy | 11-point box scale for dizziness | Range: [0-10]  0=Absence  10=Extremely severe | Baseline, 22 weeks | *Not available* | *Not available* |
|  |  |  |  |  | Safety | Adverse events | Frequency | Baseline,  6, 26 weeks | Upper respiratory tract infections (n=19/11)  Dizziness (n=17/11)  Tinnitus/tinnitus aggravated (n=5/7)  Nausea/vomiting (n=5/11)  Agitation/agitation aggravated (n=10/5)  Weight loss (n=4/8)  Headache (n=4/7) | Upper respiratory tract infections (n=18)  Dizziness (n=12)  Tinnitus/tinnitus aggravated (n=12)  Nausea/vomiting (n=8)  Agitation/agitation aggravated (n=8)  Weight loss (n=10)  Headache (n=9) |
| Napryeyenko et al. (2007) ^24^ | 2007 | Ukraine | 198 | 197 | Efficacy | 11-point box scale for dizziness | Range: [0-10]  0=Absence  10=Extremely severe | Baseline, 22 weeks | Baseline: 3.3 ± 2.3  Mean change (△): -1.7 ± 1.7 | Baseline: 3.4 ± 2.1  Mean change (△): -0.3 ± 1.1 |
|  |  |  | 200 | 200 | Safety | Adverse events | Frequency | Baseline,  6, 12, 17, 22 weeks | Headache (n=49)  Angina pectoris (n=20)  Dizziness (n=12)  Back pain (n=12)  Diarrhea (n=18)  Tinnitus (n=9)  Cough (n=14)  Influenze (n=14)  Hypertensive crisis (n=10)  Upper repiratory tract infection (n=10)  Blood pressure increased (n=4) | Headache (n=85)  Angina pectoris (n=35)  Dizziness (n=36)  Back pain (n=22)  Diarrhea (n=16)  Tinnitus (n=18)  Cough (n=11)  Influenze (n=11)  Hypertensive crisis (n=12)  Upper repiratory tract infection (n=10)  Blood pressure increased (n=13) |
| Ihl et al. (2011) ^25^ | 2011 | Ukraine | 202 | 202 | Efficacy | 11-point box scale for dizziness | Range: [0-10]  0=Absence  10=Extremely severe | Baseline, 22 weeks | Baseline: 2 ± 1.9  Mean change (△): -0.8 ± 1.45 | Baseline: 2.1 ± 1.8  Mean change (△): -0.3 ± 1.1 |
|  |  |  | 206 | 204 | Safety | Adverse events | Frequency | Baseline,  6, 12, 18, 24 weeks | Headache (n=43)  Respiratory tract infection (n=27)  Blood pressure increased, hypertension (n =22)  Dizziness (n=19)  Diarrhea (n=9)  Angina pectoris (n=6)  Tinnitus (n=2) | Headache (n=38)  Respiratory tract infection (n=20)  Blood pressure increased, hypertension (n =21)  Dizziness (n=23)  Diarrhea (n=13)  Angina pectoris (n=12)  Tinnitus (n=15) |
| Herrschaft et al. (2012) ^26^ | 2012 | Republic of Belarus, Republic of Moldova, Russian Federation | 200 | 202 | Efficacy | 11-point box scale for dizziness | Range: [0-10]  0=Absence  10=Extremely severe | Baseline, 22 weeks | Baseline: 2 ± 1.8  Mean change (△): -0.6 ± 1.09 | Baseline: 1.8 ± 1.8  Mean change (△): -0.2 ± 1.09 |
|  |  |  | 205 | 205 | Safety | Adverse events | Frequency | Baseline,  6, 12, 18, 24 weeks | Headache (n=15)  Dizziness (n=4)  Viral respiratory tract infection (n=8)  Hypertension (n=5)  Somnolence (n=8)  Upper abdominal pain (n=5) | Headache (n=17)  Dizziness (n=15)  Viral respiratory tract infection (n=6)  Hypertension (n=8)  Somnolence (n=4)  Upper abdominal pain (n=7) |
| Rina et al. (2021) ^27^ | 2021 | China | 191 | 189 | Efficacy | Traditional Chinese Medicine (TCM) | Range:  1= Ineffective  2=Effective  3=Obvious effective  4=Recovery | 2 weeks | Ineffective (n=165)  Effective (n=26)  Obvious effective (n=0)  Recovery (n=0) | Ineffective (n=167)  Effective (n=22)  Obvious effective (n=0)  Recovery (n=0) |
|  |  |  |  |  |  |  |  | 4 weeks | Ineffective (n=62)  Effective (n=119)  Obvious effective (n=9)  Recovery (n=1) | Ineffective (n=73)  Effective (n=109)  Obvious effective (n=5)  Recovery (n=2) |
|  |  |  |  |  |  |  |  | 6 weeks | Ineffective (n=14)  Effective (n=67)  Obvious effective (n=54)  Recovery (n=56) | Ineffective (n=32)  Effective (n=79)  Obvious effective (n=41)  Recovery (n=37) |
|  |  |  |  |  |  | Dizziness Handicap Inventory (DHI) | Range: [0-100]  16-34=Mild handicap  36-52=Moderate handicap  54-=Severe handicap | Baseline,  2, 4, 6 weeks | Mean change (△): -33 ± 15 | Mean change (△): -30 ± 15 |
|  |  |  |  |  |  | Visual Analogue Scale (VAS) | Range: [0-10]  0=No  10=Severe | Baseline,  2,4,6 weeks | Mean change (△): -3.8 ± 1.6 | Mean change (△): -3.2 ± 1.7 |
| Heide et al. (2022) ^28^ | 2022 | Germany | 18 | 18 | Efficacy | Visual Analogue Scale (VAS) | Range: [0-100]  0=No  100=Severe | Baseline,  120, 180 days | Baseline: 45 (95%CI: 30.0, 55.0)  120 days (△): -17.8(95%CI: -26.7, -8.8)  180 days (△): -20.4(95%CI: -30.7, -10.1) | Baseline: 27.5 (95%CI: 20.0, 40.0)  120 days (△): -3.9 (95%CI: -15.0, 7.2)  180 days (△): -6.8 (95%CI: -18.0, 4.4) |
|  |  |  | 21 | 19 | Safety | Adverse events | Frequency | Baseline, 6, 12, 18, 24 weeks | Infection with herpes zoster (n=1)  Acute pancreatitis, vertebral fracture (n=2)  Diarrhea (n=1) | Hypertensive hemorrhage in the left basal ganglia (n=1) |
| Li and Cao (2024) ^29^ | 2024 | China | 85 | 85 | Efficacy | Dizziness Handicap Inventory (DHI) | Range: [0-100]  16-34=Mild handicap  36-52=Moderate handicap  54-=Severe handicap | Baseline,  2 weeks | *Not available* | *Not available* |

Abbreviations: T, treatment group; C, control group

*Allocated to GbE 120 mg / Allocated to GbE 240 mg
